# *Ypsolophachicoi* sp. n., the second representative of the widespread micromoth genus *Ypsolopha* Latreille (Lepidoptera, Ypsolophidae) from the Andes of northern Chile

**DOI:** 10.3897/BDJ.9.e72306

**Published:** 2021-08-27

**Authors:** Héctor A. Vargas

**Affiliations:** 1 Universidad de Tarapacá, Facultad de Ciencias Agronómicas, Departamento de Recursos Ambientales, Arica, Chile Universidad de Tarapacá, Facultad de Ciencias Agronómicas, Departamento de Recursos Ambientales Arica Chile

**Keywords:** genital morphology, host plant, *
Muehlenbeckia fruticulosa
*, new species, Polygonaceae

## Abstract

**Background:**

The largest number of species of the widespread and highly diverse micromoth genus *Ypsolopha* Latreille, 1796 (Lepidoptera, Yponomeutoidea, Ypsolophidae) is known from the Northern Hemisphere. Only seven species have been described from the Neotropical Region, two of which occur in Chile.

**New information:**

The adult stage of *Ypsolophachicoi* sp. n. from the arid highlands of the western slopes of the Andes of northern Chile is described and illustrated. Its larvae feed on the native shrub *Muehlenbeckiafruticulosa* (Walp.) Standl. (Polygonaceae). The morphology of the genitalia of *Y.chicoi* sp. n. resembles that of the only congeneric known to occur in the same geographic area, *Y.moltenii* Vargas, 2018, whose larvae feed on *Adesmiaverrucosa* Meyen (Fabaceae). Besides using different host plants, the two species can be accurately separated, based on morphological differences in female and male genitalia.

## Introduction

*Ypsolopha* Latreille, 1796 (Lepidoptera, Yponomeutoidea, Ypsolophidae) is a widespread and diverse genus of micromoths with more than 160 species described (Jin et al. 2013). Larvae of *Ypsolopha* feed on a wide range of plants, partially concealed by silk webs (Dugdale et al. 1998), with specific host ranges varying from oligophagy to polyphagy (Anikin et al. 2006, Jin et al. 2013, Sohn et al. 2013, Akulov et al. 2018). The largest number of species of this genus is known from the Northern Hemisphere and additional representatives continue to be discovered there (Sohn et al. 2010, Ponomarenko and Zinchenko 2013, Na et al. 2016, Corley et al. 2019, Ponomarenko 2020, Sachkov and Zolotuhin 2020, Corley and Ferreira 2021), while the knowledge of the Neotropical fauna is currently restricted to seven described species, only two of which occur in Chile (Clarke 1965, Heppner 1984, Vargas 2018).

Despite the extreme aridity of the natural environments of the highlands of the western slopes of the Andes of the northernmost part of Chile, at about 18° S, recent surveys have revealed that their native plants harbour previously-overlooked representatives of a few micromoth families (e.g. Vargas 2019, Vargas 2020, Vargas 2021). Records of *Ypsolopha* were unknown in this area until the discovery of *Y.moltenii* Vargas, 2018, whose larvae feed on inflorescences of the shrub *Adesmiaverrucosa* Meyen (Fabaceae) (Vargas 2018). Additional adults of *Ypsolopha* were recently reared from larvae collected on another native shrub in the same area. The subsequent morphological study of their genitalia revealed that these specimens represent an undescribed species, whose description is provided here.

## Materials and methods

The examined specimens were reared from larvae collected in April 2021 on the native shrub *Muehlenbeckiafruticulosa* (Walp.) Standl. (Polygonaceae) near Socoroma Village, at about 3400 m elevation on the western slopes of the Andes of the Parinacota Province of northern Chile. The abdomen of each specimen was removed and placed in 10% potassium hydroxide (KOH) for a few minutes for genitalia dissection. Chlorazol black and Eosin Y were used to stain the genitalia previous to mounting on slides with Euparal. The length of the anterior apophysis was measured from the anterior edge of tergum VIII. The images of the adult and the genitalia were captured with a Sony CyberShot DSC-HX200V digital camera, attached to a Leica M125 stereomicroscope and a Micropublisher 3.3 RTV-QImaging digital camera, attached to an Olympus BX51. The distribution map was generated using SimpleMappr (Shorthouse 2010). The pinned specimens and their genitalia slides are deposited in the “Colección Entomológica de la Universidad de Tarapacá” (IDEA), Arica, Chile.

## Taxon treatments

### Ypsolopha chicoi

Vargas sp. n.

72DC3684-B710-5206-94BE-5520841984BF

54EF6F5A-A07A-499E-BB1E-0229073FCE96

#### Materials

- **Type status:**Holotype. **Occurrence:** sex: male; otherCatalogNumbers: IDEA-LEPI-2021-001, genitalia slide HAV-1471; **Taxon:** order: Lepidoptera; family: Ypsolophidae; genus: Ypsolopha; specificEpithet: chicoi; taxonRank: species; nomenclaturalCode: ICZN; **Location:** continent: South America; country: Chile; stateProvince: Parinacota; locality: About 2 km south of Socoroma village; verbatimElevation: 3400 m; verbatimLatitude: 18°27'22"S; verbatimLongitude: 69°35'15"W; **Identification:** identifiedBy: Héctor A. Vargas; dateIdentified: June 2021; **Event:** samplingProtocol: One male adult emerged May 2021, reared from larva collected on *Muehlenbeckiafruticulosa* in April 2021; year: 2021; verbatimEventDate: May 2021; **Record Level:** type: PhysicalObject; language: en; institutionCode: IDEA; basisOfRecord: PreservedSpecimen
- **Type status:**Paratype. **Occurrence:** sex: male; otherCatalogNumbers: IDEA-LEPI-2021-002, genitalia slide HAV-1458; **Taxon:** order: Lepidoptera; family: Ypsolophidae; genus: Ypsolopha; specificEpithet: chicoi; taxonRank: species; nomenclaturalCode: ICZN; **Location:** continent: South America; country: Chile; stateProvince: Parinacota; locality: About 2 km south of Socoroma village; verbatimElevation: 3400 m; verbatimLatitude: 18°27'22"S; verbatimLongitude: 69°35'15"W; **Identification:** identifiedBy: Héctor A. Vargas; dateIdentified: June 2021; **Event:** samplingProtocol: One male adult emerged May 2021, reared from larva collected on *Muehlenbeckiafruticulosa* in April 2021; year: 2021; verbatimEventDate: May 2021; **Record Level:** type: PhysicalObject; language: en; institutionCode: IDEA; basisOfRecord: PreservedSpecimen
- **Type status:**Paratype. **Occurrence:** sex: male; otherCatalogNumbers: IDEA-LEPI-2021-003, genitalia slide HAV-1460; **Taxon:** order: Lepidoptera; family: Ypsolophidae; genus: Ypsolopha; specificEpithet: chicoi; taxonRank: species; nomenclaturalCode: ICZN; **Location:** continent: South America; country: Chile; stateProvince: Parinacota; locality: About 2 km south of Socoroma village; verbatimElevation: 3400 m; verbatimLatitude: 18°27'22"S; verbatimLongitude: 69°35'15"W; **Identification:** identifiedBy: Héctor A. Vargas; dateIdentified: June 2021; **Event:** samplingProtocol: One male adult emerged May 2021, reared from larva collected on *Muehlenbeckiafruticulosa* in April 2021; year: 2021; verbatimEventDate: May 2021; **Record Level:** type: PhysicalObject; language: en; institutionCode: IDEA; basisOfRecord: PreservedSpecimen
- **Type status:**Paratype. **Occurrence:** sex: female; otherCatalogNumbers: IDEA-LEPI-2021-004, genitalia slide HAV-1459; **Taxon:** order: Lepidoptera; family: Ypsolophidae; genus: Ypsolopha; specificEpithet: chicoi; taxonRank: species; nomenclaturalCode: ICZN; **Location:** continent: South America; country: Chile; stateProvince: Parinacota; locality: About 2 km south of Socoroma village; verbatimElevation: 3400 m; verbatimLatitude: 18°27'22"S; verbatimLongitude: 69°35'15"W; **Identification:** identifiedBy: Héctor A. Vargas; dateIdentified: June 2021; **Event:** samplingProtocol: One female adult emerged May 2021, reared from larva collected on *Muehlenbeckiafruticulosa* in April 2021; year: 2021; verbatimEventDate: May 2021; **Record Level:** type: PhysicalObject; language: en; institutionCode: IDEA; basisOfRecord: PreservedSpecimen
- **Type status:**Paratype. **Occurrence:** sex: female; otherCatalogNumbers: IDEA-LEPI-2021-005, genitalia slide HAV-1461; **Taxon:** order: Lepidoptera; family: Ypsolophidae; genus: Ypsolopha; specificEpithet: chicoi; taxonRank: species; nomenclaturalCode: ICZN; **Location:** continent: South America; country: Chile; stateProvince: Parinacota; locality: About 2 km south of Socoroma village; verbatimElevation: 3400 m; verbatimLatitude: 18°27'22"S; verbatimLongitude: 69°35'15"W; **Identification:** identifiedBy: Héctor A. Vargas; dateIdentified: June 2021; **Event:** samplingProtocol: One female adult emerged May 2021, reared from larva collected on *Muehlenbeckiafruticulosa* in April 2021; year: 2021; verbatimEventDate: May 2021; **Record Level:** type: PhysicalObject; language: en; institutionCode: IDEA; basisOfRecord: PreservedSpecimen
- **Type status:**Paratype. **Occurrence:** sex: female; otherCatalogNumbers: IDEA-LEPI-2021-006, genitalia slide HAV-1472; **Taxon:** order: Lepidoptera; family: Ypsolophidae; genus: Ypsolopha; specificEpithet: chicoi; taxonRank: species; nomenclaturalCode: ICZN; **Location:** continent: South America; country: Chile; stateProvince: Parinacota; locality: About 2 km south of Socoroma village; verbatimElevation: 3400 m; verbatimLatitude: 18°27'22"S; verbatimLongitude: 69°35'15"W; **Identification:** identifiedBy: Héctor A. Vargas; dateIdentified: June 2021; **Event:** samplingProtocol: One female adult emerged May 2021, reared from larva collected on *Muehlenbeckiafruticulosa* in April 2021; year: 2021; verbatimEventDate: May 2021; **Record Level:** type: PhysicalObject; language: en; institutionCode: IDEA; basisOfRecord: PreservedSpecimen

#### Description

**Figure 1.**
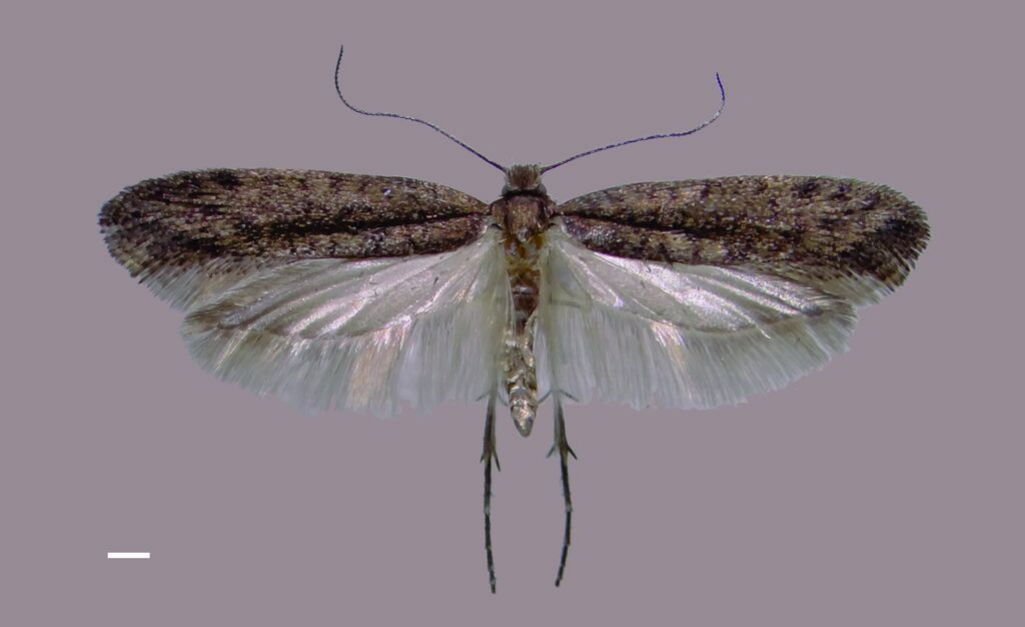
Male holotype of *Ypsolophachicoi* sp. n. in dorsal view. Scale bar 1 mm.

Male (Fig. 1). Forewing length 9.1–9.4 mm.

Head. Vertex with elongated narrow scales with slightly cleft apex, mostly greyish-brown, a few yellowish-white scattered near posterior margin; frons greyish-brown, mostly appressed scales, elongated narrow scales with slightly cleft yellowish-white apex laterally. Antenna about 2/3 length of forewing; mostly greyish-brown, a few yellowish-grey scales on scape. Ocellus posterior to scape base. Maxillary palpus with greyish-brown scales with yellowish-white apex. Labial palpus mostly greyish-brown; second segment with erect scales with yellowish-white apex projected to nearly half of third segment; third segment appressed scaled; inner face of first segment and basal part of second segment yellowish-white.

Thorax. Greyish-brown dorsally; mostly yellowish-white laterally with a few greyish-brown scattered scales. Foreleg mostly greyish-brown with yellowish-white scattered scales, tibial epiphysis greyish-brown. Mid-leg similar to foreleg in colouration, tibial spurs greyish-brown. Hindleg mostly yellowish-white with greyish-brown scattered scales, tibial spurs and tarsus mostly greyish-brown.

Abdomen. Mostly yellowish-white with a few greyish-brown scattered scales. Male tergum VIII triangular, with a pair of sclerotised posterior projections encircling the base of pleural lobes; sternum VIII mostly membranous, with a pair of coremata.

**Figure 2.**
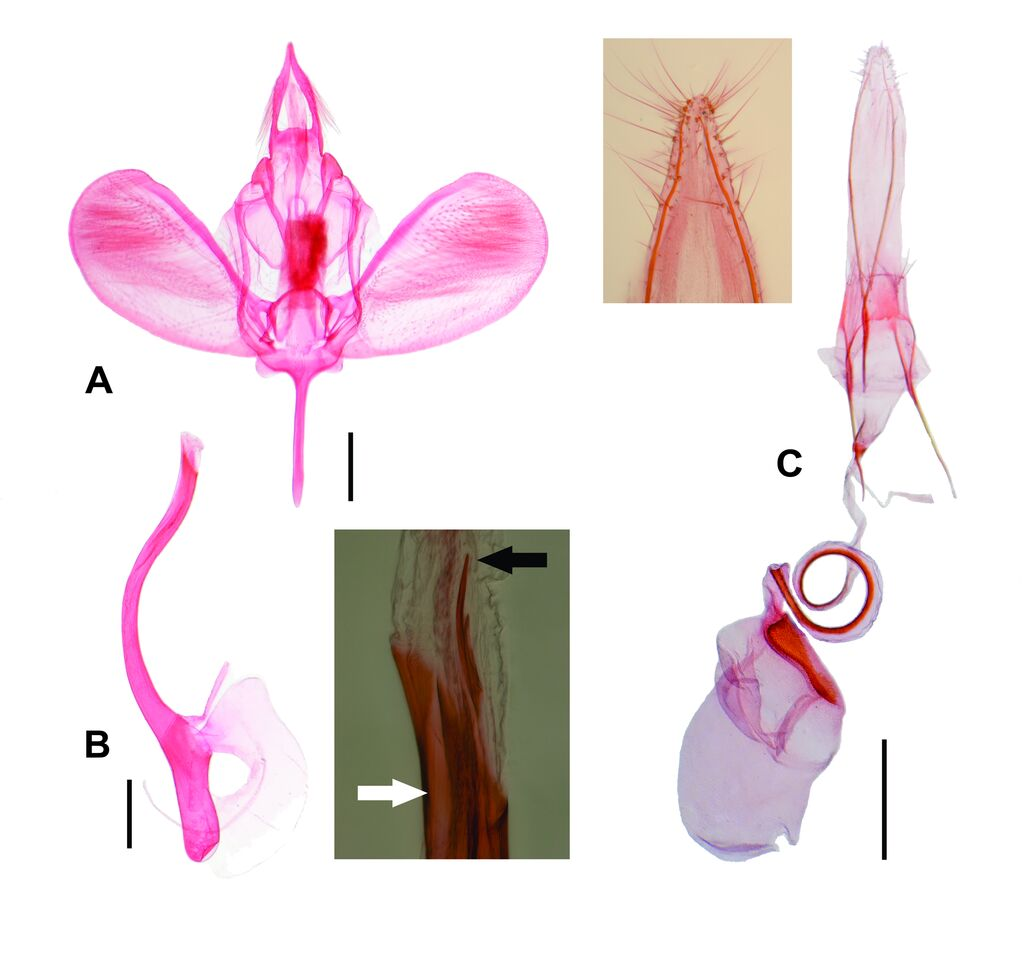
Genitalia of *Ypsolophachicoi* sp. n. A. Male genitalia in ventral view, phallus removed. B. Phallus in lateral view. C. Female genitalia in ventral view. Upper rectangle shows a detail of the papillae analis in ventral view. Bottom rectangle shows the apex of phallus with the vesica and cornuti in lateral view; black arrow indicates apex of the longest cornutus; white arrow indicates ventral cleft. Scale bars 0.2, 0.2 and 0.5 mm, respectively.

Male genitalia (Fig. 2)

Tegumen bilobed, anterior margin deeply excavated. Socius digitate, narrowing apically, hair-like scales on the medial third. Gnathos Y-shaped; ventral arm widened, flattened, coarse, round apex, length about half the dorsal arms. Saccus cylindrical, length about 1.5 times the socius, round apex. Anellus with two narrow longitudinal sclerotised stripes separated by membranous area. Valva ovate; costal margin slightly rounded, with a slightly differentiated knob on the apex; distal margin broadly rounded. Phallus sub-cylindrical, broadly ventrally curved at middle, apex with a narrow ventral cleft, coecum about a fourth the phallus length; vesica with two small spine-shaped cornuti, length of the longest about twice the width of the distal third of the phallus.

Female. Similar to male in size and maculation.

Female genitalia (Fig. 2)

Papillae analis narrow, elongated, slightly sclerotised, with few hair-like setae. Posterior apophysis spine-shaped, about four times the length of papillae analis. Anterior apophysis spine-shaped, about 2/3 the length of posterior apophysis, base bifurcated, dorsal arm continuous with tergum VIII, ventral arm continuous with sternum VIII. Sternum VIII rectangular, lateral margins slightly concave, elongated hair-like setae on posterior vertices. Antrum membranous, cone-shaped, with a sclerotised ring. Ductus bursae mainly membranous, coiled, a narrow longitudinal sclerotised patch about twice the length of anterior apophysis. Corpus bursae mainly membranous, about 2/3 the length of ductus bursae, signum on the basal half of the right side of corpus bursae, as a minutely sculptured plate, about 1/4 of length of the sclerotised patch of ductus bursae. Ductus seminalis at base of ductus bursae.

#### Diagnosis

*Ypsolophachicoi* sp. n. is recognisable, based on the morphology of the genitalia. The saccus reaches about 1.5 times the socius length, the phallus reaches about four times the coecum length and has a narrow ventral cleft at apex and the vesica has two small spine-shaped cornuti, the longest of which reaches about twice the width of the distal third of the phallus in the male and the sclerotised patch of the ductus bursae reaches about four times the length of the signum and about twice the length of the anterior apophysis in the female. The morphology of the genitalia of *Y.chicoi* sp. n. is remarkably similar to that of *Y.moltenii*, the only congeneric previously known from the same locality. However, in the male of *Y.moltenii*, the saccus and socius are similar in length, the phallus reaches about 3.3 times the coecum length and lacks a ventral cleft at apex and the vesica has a long arrow-shaped cornutus, whose length reaches about three times the width of the distal third of the phallus and, in the female, the sclerotised patch of the ductus bursae is slightly longer than the signum and anterior apophysis. Besides the morphological differences, the two species are associated with plants of different families: *Y.chicoi* sp. n. with Polygonaceae and *Y.moltenii* with Fabaceae.

#### Etymology

*Ypsolophachicoi* sp. n. is named in honour of the eminent Brazilian musician and composer Chico Buarque (Francisco Buarque de Hollanda), for all his wonderful contribution to the “Música Popular Brasileira”.

#### Distribution

**Figure 3.**
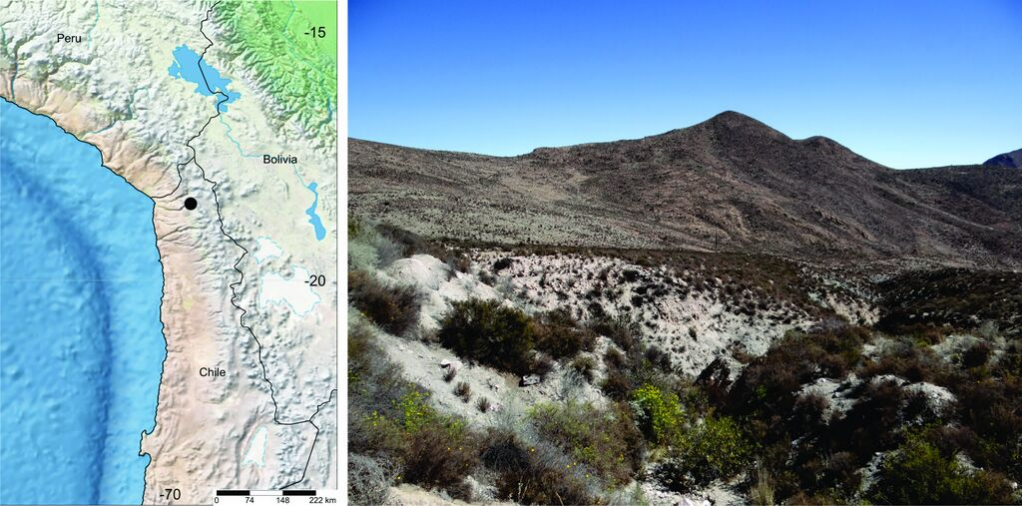
Geographic distribution and habitat of *Ypsolophachicoi* sp. n. Rectangle on left shows the type locality (black circle) in northern Chile. Rectangle on right shows the habitat in the type locality, near the Socoroma Village, at about 3400 m elevation in the Andes.

*Ypsolophachicoi* sp. n. is known only from the type locality, near Socoroma Village, on the western slopes of the Andes of the Parinacota Province of northern Chile (Fig. 3).

#### Biology

Larvae of *Y.chicoi* sp. n. feed partially concealed in silk webs on leaves, buds, flowers and fruits of the native shrub *Muehlenbeckiafruticulosa* (Walp.) Standl. (Polygonaceae). This shrub is also found in Bolivia and Peru; its Chilean range is restricted to a belt at about 3400–3500 m elevation in the northernmost part of the country (Rodriguez et al. 2018).

## Discussion

Recent contributions suggest that the taxonomic diversity and natural history of Yponomeutoidea remain underexplored in the Neotropics (e.g. Becker 2009, Becker 2013, Moreira et al. 2019). In the present study, a single field trip was enough to discover a previously-unknown species and to unveil a novel host plant association for the Neotropical fauna of *Ypsolopha*.

With the discovery of *Y.chicoi* sp. n., three species of *Ypsolopha* are now known from Chile. It is remarkable that the only two species currently recorded in the mainland part of the country (*Y.moltenii* and *Y.chicoi* sp. n.) occur in the same extremely arid area. However, this pattern certainly reflects a sampling bias. As larvae of *Ypsolopha* are phytophagous, surveys on environments with higher plant diversity in central and south Chile, where additional representatives of Yponomeutoidea have been recently discovered (Beche and Parra 1998, Sohn and Alba 2014, Cepeda 2016), should reveal a higher diversity of this micromoth genus.

Shrubs and trees of a wide range of genera and families have been recorded as hosts of *Ypsolopha* in the Northern Hemisphere (Anikin et al. 2006, Robinson et al. 2010, Jin et al. 2013, Akulov et al. 2018). Host records in the Neotropics are restricted to Ephedraceae (*Ephedraamericana* Humb. & Bonpl. ex Willd.) for *Y.cordillerella* (Kieffer & Jörgensen, 1910) and Fabaceae for *Y.moltenii* (Kieffer and Jörgensen 1910, Vargas 2018). Feeding by *Y.chicoi* sp. n. on *M.fruticulosa* represents the first record of the family Polygonaceae and *Muehlenbeckia*, a genus with Gondwanan distribution (Schuster et al. 2013), as host of a Neotropical species of *Ypsolopha*.

Additional surveys for adults and larvae of *Ypsolopha* in different environments of South America are encouraged, not only to collect unknown species, but also to improve the knowledge of the already-described ones, as only one sex is known for many of them, all remain known only from their respective type localities and their host plants are poorly documented (e.g. Kieffer and Jörgensen 1910, Clarke 1965, Vargas 2018). An improvement in the knowledge of the taxonomic diversity and natural history is needed to understand the evolutionary patterns of the Neotropical species of *Ypsolopha*.

## Supplementary Material

XML Treatment for Ypsolopha chicoi
